# EmrR-Dependent Upregulation of the Efflux Pump EmrCAB Contributes to Antibiotic Resistance in *Chromobacterium violaceum*

**DOI:** 10.3389/fmicb.2018.02756

**Published:** 2018-11-15

**Authors:** Kelly C. M. Barroso, Maristela Previato-Mello, Bianca B. Batista, Juliana H. Batista, José F. da Silva Neto

**Affiliations:** Departamento de Biologia Celular e Molecular e Bioagentes Patogênicos, Faculdade de Medicina de Ribeirão Preto, Universidade de São Paulo, São Paulo, Brazil

**Keywords:** *Chromobacterium violaceum*, MarR transcription factors, antibiotic resistance, quinolone resistance, drug efflux pumps, quorum sensing, violacein

## Abstract

*Chromobacterium violaceum* is an environmental Gram-negative bacterium that causes infections in humans. Treatment of *C. violaceum* infections is difficult and little is known about the mechanisms of antibiotic resistance in this bacterium. In this work, we identified mutations in the MarR family transcription factor EmrR and in the protein GyrA as key determinants of quinolone resistance in *C. violaceum*, and we defined EmrR as a repressor of the MFS-type efflux pump EmrCAB. Null deletion of *emrR* caused increased resistance to nalidixic acid, but not to other quinolones or antibiotics of different classes. Moreover, the Δ*emrR* mutant showed decreased production of the purple pigment violacein. Importantly, we isolated *C. violaceum* spontaneous nalidixic acid-resistant mutants with a point mutation in the DNA-binding domain of EmrR (R92H), with antibiotic resistance profile similar to that of the Δ*emrR* mutant. Other spontaneous mutants with high MIC values for nalidixic acid and increased resistance to fluoroquinolones presented point mutations in the gene *gyrA*. Using DNA microarray, Northern blot and EMSA assays, we demonstrated that EmrR represses directly a few dozen genes, including the *emrCAB* operon and other genes related to transport, oxidative stress and virulence. This EmrR repression on *emrCAB* was relieved by salicylate. Although mutation of the *C. violaceum emrCAB* operon had no effect in antibiotic susceptibility or violacein production, deletion of *emrCAB* in an *emrR* mutant background restored antibiotic susceptibility and violacein production in the Δ*emrR* mutant. Using a biosensor reporter strain, we demonstrated that the lack of pigment production in Δ*emrR* correlates with the accumulation of quorum-sensing molecules in the cell supernatant of this mutant strain. Therefore, our data revealed that overexpression of the efflux pump EmrCAB via mutation and/or derepression of EmrR confers quinolone resistance and alters quorum-sensing signaling in *C. violaceum*, and that point mutation in *emrR* can contribute to emergence of antibiotic resistance in bacteria.

## Introduction

Antibiotic resistance is a global public health problem with high impact on the treatment of bacterial infections, as many multidrug-resistant (MDR) strains have evolved in clinically relevant pathogens (Davies and Davies, 2010; Rossolini et al., 2014). Several mechanisms can mediate intrinsic and acquired resistance, including antibiotic removal by membrane efflux pumps, inactivation of the antibiotic, modification of the antibiotic target, and preventing the entrance of antibiotics into the cell (Cox and Wright, 2013; Blair et al., 2015). In most cases, antibiotic-resistant strains arise by mutations in transcription factors that regulate the genes involved in antibiotic resistance. For instance, a common pathway that leads to overexpression of efflux pumps in MDR bacteria is the occurrence of mutations in transcription factors that regulate the genes encoding these efflux pumps (Fernandez and Hancock, 2012; Blair et al., 2015).

Members of the multiple antibiotic resistance regulator (MarR) family of transcription factors are involved in several biological processes in bacteria, including oxidative stress, virulence, and antibiotic resistance; most of them act as transcription repressors (Deochand and Grove, 2017). The first described member of this family, the *marR* gene of *Escherichia coli*, represses the *marRAB* operon, involved in resistance to multiple antibiotics (George and Levy, 1983; Seoane and Levy, 1995). Other MarR family transcription factors have been described regulating efflux pumps that contribute to antibiotic resistance, such as MexR in *Pseudomonas aeruginosa* (Chen et al., 2008), MepR in *Staphylococcus aureus* (Birukou et al., 2013), MarR in *Mycobacterium smegmatis* (Zhang et al., 2014), and EmrR in *E. coli* (Lomovskaya and Lewis, 1992). Mutation in *emrR*, or compounds such as salicylate, disrupt the repression of EmrR on the operon *emrRAB*, allowing expression of the major facilitator superfamily (MFS) efflux pump EmrAB, which exports hydrophobic compounds such as the antibiotic nalidixic acid (Lomovskaya and Lewis, 1992; Xiong et al., 2000). It has been suggested that EmrAB could form a tripartite pump with the outer membrane protein TolC (Tanabe et al., 2009). Although a similar mechanism has been described controlling the EmrCABsm efflux pump in *Stenotrophomonas maltophilia* (Huang et al., 2013), it is still unclear whether antibiotic resistance can emerge as a consequence of point mutation in the *emrR* gene, and other genes regulated by EmrR have not been identified.

*Chromobacterium violaceum*, a Gram-negative free-living, saprophytic bacterium found in waters and soils of tropical and subtropical regions, is an opportunistic pathogen that causes infections with rapid dissemination and high mortality (Yang and Li, 2011; Batista and da Silva Neto, 2017). It produces the microbicidal purple pigment violacein, whose synthesis is activated by the *N*-acyl-L-homoserine lactone (AHL)-based quorum-sensing system CviI/CviR (Stauff and Bassler, 2011; Durán et al., 2016). Thus, deletion of the AHL-synthase gene *cviI* generates a biosensor strain that produce violacein only when exogenous AHL molecules are provided (Morohoshi et al., 2008). Although infection by *C. violaceum* in the hospital environment is uncommon, cases of nosocomial pneumonia caused by *C. violaceum* have been reported in patients in intensive care units (Hagiya et al., 2014). Failure in treating *C. violaceum* infections relates to resistance to some antibiotics, especially β-lactams, but this bacterium is susceptible to quinolones and carbapenems (Aldridge et al., 1988; Yang and Li, 2011; Hagiya et al., 2014; Madi et al., 2015). Recently, it has been suggested that class A β-lactamases (KPC) evolved from the *Chromobacterium* genus (Gudeta et al., 2016). Investigating the regulatory mechanisms that control antibiotic resistance in *C. violaceum* is key to understanding how resistance to currently useful antibiotics can emerge under conditions such as those found during the treatment of *C. violaceum* infections. In this work, we identified the MarR family transcription factor EmrR as a regulator of antibiotic resistance in *C. violaceum*. We defined the repertoire of genes regulated by EmrR, and characterized both null and spontaneous *emrR* mutants by antimicrobial susceptibility assays.

## Materials and Methods

### Bacterial Strains, Growth Conditions, Plasmids, and Oligonucleotides

Bacterial strains and plasmids used in this work are listed in Table 1. Mutant strains of *C. violaceum* were derived from the wild-type strain ATCC 12472 (Vasconcelos et al., 2003). All bacterial strains of *C. violaceum* and *E. coli* were grown at 37°C in Luria-Bertani (LB) medium. Antibiotic susceptibility assays were performed on Mueller-Hinton (MH) medium (Sigma). For plasmid selection in general cloning procedures, LB medium was supplemented with the antibiotics tetracycline (10 μg/ml), kanamycin (50 μg/ml), and ampicillin (100 μg/ml). Oligonucleotide sequences are listed in Supplementary Table S1.

**Table 1:** Bacterial strains and plasmids used in this work.

Strain or plasmid	Description	Reference or source
*Escherichia coli strains*		
DH5α	Strain for cloning purposes	Hanahan, 1983
S17-1	Strain for plasmid mobilization	Simon et al., 1983
BL21 (DE3)	Strain for protein expression	Novagen
*Chromobacterium violaceum strains*		
ATCC 12472	^a^Sequenced genome (wild type)	Vasconcelos et al., 2003
JF0769	ATCC 12472, Null deletion CV_0769 (Δ*emrR*)	This work
JF07666768	ATCC 12472, Null deletion CV_0766-0767-0768 (Δ*emrCAB*)	This work
JF0766676869	ATCC 12472, Null deletion CV_0766-0767-0768-0769 (Δ*emrRCAB*)	This work
JF4091	ATCC 12472, Null deletion CV_4091 (Δ*cviI*)	This work
JF0769PM	ATCC 12472, Point mutation CV_0769 (*emrR*_R92H_)	This work
JF2298PM	ATCC 12472, Point mutation CV_2298 (*gyrA*_T85I_)	This work
JF0769(pMR*emrR*)	Δ*emrR*, complementation with *emrR*	This work
JF0769PM(pMR*emrR*)	*emrR*_R92H_, complementation with *emrR*	This work
Plasmids		
pNPTS138	Suicide vector containing *oriT sacB*; Kan^r^	D. Alley
pET-15b	His-tagged protein expression vector; Amp^r^	Novagen
pMR20	Low-copy-number and broad-host-range vector; *oriT*; Tet^r^	Roberts et al., 1996
pNPTΔ*emrR*	In frame null deletion of *emrR*	This work
pNPTΔ*emrCAB*	In frame null deletion of *emrCAB*	This work
pNPTΔ*emrRCAB*	In frame null deletion of *emrRCAB*	This work
pNPTΔ*cviI*	In frame null deletion of *cviI*	This work
pET-*emrR*	Overexpression of *emrR*	This work
pMR-*emrR*	Complementation of *emrR* mutant strains.	This work

^a^*Reference sequence according to annotated in the genome of *C. violaceum* ATCC 12472 (Vasconcelos et al., 2003)*.

### Construction and Complementation of Mutant Strains

The in-frame *emrR*, *emrCAB*, *emrRCAB*, and *cviI* gene deletions were constructed by a two-step allelic exchange procedure with the vector pNPTS138 as previously described (da Silva Neto et al., 2012). The resulting mutant strains Δ*emrR*, Δ*emrCAB*, Δ*emrRCAB*, and Δ*cviI* were confirmed by PCR using specific primers (Supplementary Table S1). For complementation of *emrR* mutants, a DNA fragment containing the full *emrR* gene was cloned into the vector pMR20 and this construct was introduced into the mutant strains by conjugation (da Silva Neto et al., 2012).

### Selection of Spontaneous Nalidixic Acid-Resistant Mutants

Selection was performed by plating 500 μl of an LB-grown overnight culture of *C. violaceum* ATCC 12472 on LB plates with increased concentrations of nalidixic acid (0.5–64 μg/ml; 1–7 times the MIC). Several spontaneous resistant colonies that appeared on these plates after incubation at 37°C for 24 h were cultivated in LB without antibiotic. After testing these colonies for nalidixic acid susceptibility (see item MIC on LB plates), resistant colonies (4 μg/ml to 512 μg/ml) were selected for sequencing the full *emrR* and the QRDR region of *gyrA*, using specific primers (Supplementary Table S1). For Sanger DNA sequencing, the amplicons of *emrR* and *gyrA* were obtained by colony PCR and sequenced in both strands using BigDye Terminator V3.1 (Applied Biosystems).

### Antibiogram by Disk Diffusion Assays

Disk diffusion assays were performed as recommended by the (Clinical and Laboratory Standards Institute [CLSI], 2013). Briefly, *C. violaceum* strains, grown on MH plates for 20 h, were resuspended in sterile saline and adjusted to 0.5 MacFarland turbidity standard. These suspensions were seeded onto MH plates using a sterile swab. On the surface of seeded plates were placed disks impregnated with 24 antibiotics (BD BBL^TM^ Sensi-Disc^TM^ Antimicrobial Susceptibility Test Discs) (Supplementary Table S2). The growth inhibition halos were recorded after 24 h incubation at 37°C. Disk diffusion assays were performed at least in biological triplicate.

### MIC Assays

MICs of nalidixic acid, kanamycin, streptomycin, tetracycline, doxycycline, erythromycin, chloramphenicol, and cefotaxime for each *C. violaceum* strain were determined by a broth macrodilution method according to CLSI guidelines (Clinical and Laboratory Standards Institute [CLSI], 2013). The MIC values were determined as the lowest concentration of the antibiotic that inhibited visible bacterial growth after incubation of the cultures in MH broth for 24 h at 37°C under shaking.

### MIC on LB Plates

The strains were grown on LB plates for 24 h, and streaked on LB plates with increasing concentrations of nalidixic acid (0.5, 1.0, and 2.0 μg/ml, for *emrRCAB* mutants). The susceptibility profiles of the spontaneous nalidixic acid-resistant mutants were determined using this method (1 μg/ml to 512 μg/ml of nalidixic acid). After 24 h incubation at 37°C, the bacterial growth was recorded.

### Detection of Extracellular Quorum-Sensing Molecules

The presence of *N*-acyl-L-homoserine lactones (AHLs) in the culture supernatant of *C. violaceum* strains was evaluated by the production of violacein in the biosensor strain Δ*cviI*, using an agar plate assay, as previously described (Morohoshi et al., 2008). Briefly, filtered supernatants (80 μl) of overnight cultures were added inside wells sunken on LB agar plates soaked with the Δ*cviI* strain. After 48 h incubation at 25°C, the appearance of a violacein halo was recorded on the plates. Biofilm formation was assayed on polypropylene tubes with the strains cultured in LB medium for 16 h under static conditions, using the crystal violet method (Azeredo et al., 2017).

### RNA Isolation

*Chromobacterium violaceum* strains were grown at 37°C in LB until mid-log phase (OD_600_ of 0.8–1.0). After cell harvesting by centrifugation, the total RNA was extracted with TRIzol reagent (Ambion), and purified with the illustra RNAspin Mini RNA isolation kit (GE Healthcare), which includes a DNase treatment step. To test *emrCAB*-inducing conditions by Northern blot, the wild-type strain ATCC 12472 was grown at 37°C in LB until OD_600_ of 0.8. Then, the culture was split into four aliquots and either left untreated or treated for 10 min with salicylate (0.1, 1, and 10 mM), nalidixic acid (0.1, 0.2, and 0.5 mM), or ethidium bromide (0.1, 0.2, and 0.5 mM), and the cells were used for RNA extraction. RNA concentration was determined with a NanoDrop spectrophotometer (Thermo Scientific) and RNA integrity was checked by using formaldehyde-denaturing agarose gels.

### DNA Microarray Analysis

A detailed description of the custom-designed oligonucleotide microarray slides (Agilent Technologies) was published previously (Previato-Mello et al., 2017). All procedures for cRNA labeling, hybridization, and washing of the slides as well as data acquisition, extraction, and normalization were performed exactly as described (Previato-Mello et al., 2017), and following manufacturer’s instructions (Agilent Technologies). Data sets included three independent biological experiments with RNA extracted from *C. violaceum* ATCC 12472 and Δ*emrR* strains grown in LB at 37°C until mid-log phase as stated above. Differentially expressed genes were those that had their expression levels altered at least two-fold (Δ*emrR*/WT).

### Accession Number(s)

Microarray raw data have been deposited in the Gene Expression Omnibus (GEO) database^1^ with accession number GSE112521.

### Northern Blot Analysis

Samples of total RNA (7 μg) extracted as stated above were used for Northern blot assays as previously described (da Silva Neto et al., 2012; Previato-Mello et al., 2017). Specific probes for each indicated gene were amplified by PCR (primers listed in Supplementary Table S1), and labeled with [α-^32^P]dCTP (PerkinElmer) by using an Exo-Klenow enzyme DECAprime II kit (Ambion). After membrane hybridization in ULTRAhyb buffer (Ambion) and washing, the signal was detected by autoradiography.

### Expression and Purification of EmrR

The coding region of the *C. violaceum emrR* gene (CV_0769) was amplified by PCR using specific primers (Supplementary Table S1), and cloned into the vector pET15b as a 501-pb NdeI/BamHI DNA fragment. The recombinant His-EmrR protein was produced in *E. coli* BL21(DE3) after induction with 1 mM IPTG, and purified by NTA-resin affinity chromatography (Qiagen) as previously described (da Silva Neto et al., 2012).

### Western Blot

Proteins from total extracts of *C. violaceum* strains were separated on 15% SDS-PAGE gels, and transferred onto a nitrocellulose membrane (Amershan Protran). Membranes were blocked and incubated with a 1:1,000 dilution of anti-EmrR mouse polyclonal antiserum. After membrane incubation with a secondary anti-mouse IgG conjugated to peroxidase, detection was performed using the LumiGLO western blotting protein detector kit as recommended by the manufacturer (KPL). The polyclonal anti-EmrR antibody was developed in mice according to an experimental protocol approved by the Local Ethical Animal Committee (CEUA) of FMRP-USP (protocol number 147/2014).

### Electrophoretic Mobility Shift Assay (EMSA)

DNA probes corresponding to promoter regions of indicated genes were amplified by PCR from the ATCC 12472 genome, using specific primers (Supplementary Table S1). These DNA fragments were end labeled with [γ-^32^P]ATP (PerkinElmer) by using T4 polynucleotide kinase (Thermo Scientific) and purified with the Wizard^®^ SV gel and PCR clean-up system (Promega). The DNA binding reactions containing the DNA probes and different amounts of DTT-reduced His-EmrR were performed in an interaction buffer, as previously described (da Silva Neto et al., 2012; Previato-Mello et al., 2017).

## Results

### Null Deletion of *emrR* Confers Resistance to Nalidixic Acid

**FIGURE 1:**
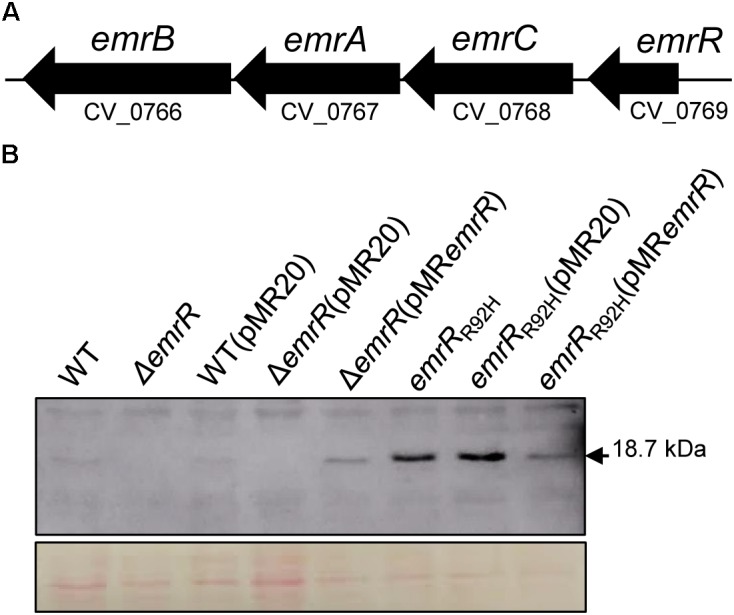
Genomic analysis of the *emrR*/*emrCAB* cluster and molecular characterization of *emrR* mutant strains. **(A)** Scheme showing that the gene *emrR* is located near to the operon *emrCAB* on the chromosome of *C. violaceum*. **(B)** Western blot confirmed that deletion of the *emrR* gene abolished production of the EmrR protein, while point mutation in *emrR* resulted in an EmrR variant unable to autorepress its own production. Levels of EmrR in the indicated *C. violaceum* strains detected with anti-EmrR antiserum. Equal protein loading determined by membrane staining with Ponceau S (bottom panel).

**FIGURE 2:**
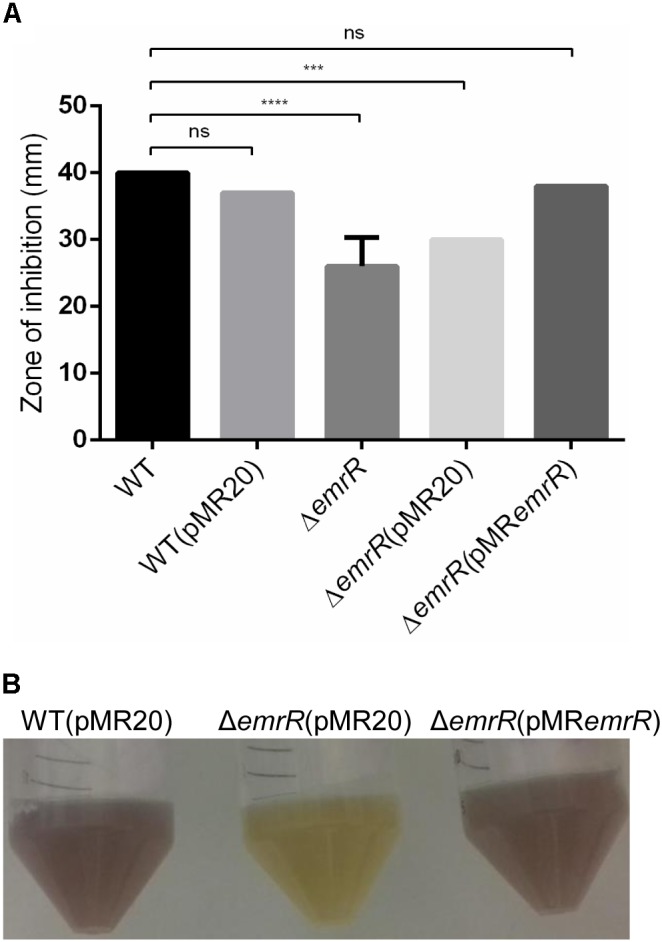
Null deletion of *emrR* confers increased resistance to nalidixic acid and decreased production of violacein. **(A)** Disk diffusion assays for nalidixic acid performed on MH plates in biological triplicate with the indicated *C. violaceum* strains. The error bars indicate standard deviations. **(B)** Decreased production of violacein in the null mutant *emrR*. The indicated strains were grown in LB broth at 37°C for 24 h. *P*-values were determined by one-way ANOVA Tukey’s multiple comparisons test: ^∗∗∗∗^*P* < 0.0001; ^∗∗∗^*P* = 0.0007.

In the genome of *C. violaceum* ATCC 12472 there are at least 15 genes encoding MarR family transcription factors (Vasconcelos et al., 2003), but only *ohrR* has been characterized (da Silva Neto et al., 2012; Previato-Mello et al., 2017). Analysis of sequence alignment and genomic position indicated that CV_0769 and the nearby genes CV_0768, CV_0767, and CV_0766 resemble the *emrR*, *emrCAB* cluster (Figure 1A) described in other bacteria, which encode the MarR family transcription factor EmrR and the MFS-type efflux pump EmrCAB (Lomovskaya and Lewis, 1992; Huang et al., 2013). To define the role of EmrR in antibiotic resistance, we constructed and characterized an *emrR* null mutant strain by antimicrobial susceptibility assays. Antibiogram tests with 24 antibiotics (Supplementary Figure S1) and determination of MIC with eight antibiotics (Supplementary Table S3) revealed that the Δ*emrR* mutant showed increased resistance specifically to nalidixic acid, a quinolone (a 13-mm decrease in the halo and a fourfold increase in the MIC). After complementation of the Δ*emrR* mutant (Figure 1B), this increased resistance to nalidixic acid was reverted, as determined by disk diffusion (Figure 2A), and MIC (Table 2). A phenotype of decreased violacein production, verified for the Δ*emrR* mutant in LB liquid cultures, was also complemented (Figure 2B). These results indicate that EmrR controls antibiotic resistance and pigment production in *C. violaceum*.

**Table 2:** MIC of indicated strains using nalidixic acid.

Strain	MIC (μg/ml)^a^
	Nalidixic acid
ATCC 12472	16
Δ*emrR*	64
ATCC 12472(pMR20)	16
Δ*emrR*(pMR20)	64
Δ*emrR*(pMR*emrR*)	16
*emrR*_R92H_	64
Δ*emrCAB*	16
Δ*emrRCAB*	16

^a^*MIC values were determined by broth macrodilution method using MH medium. These assays were performed using at least three biological replicates*.

### Point Mutations in Both EmrR and GyrA Confer Nalidixic Acid Resistance

**FIGURE 3:**
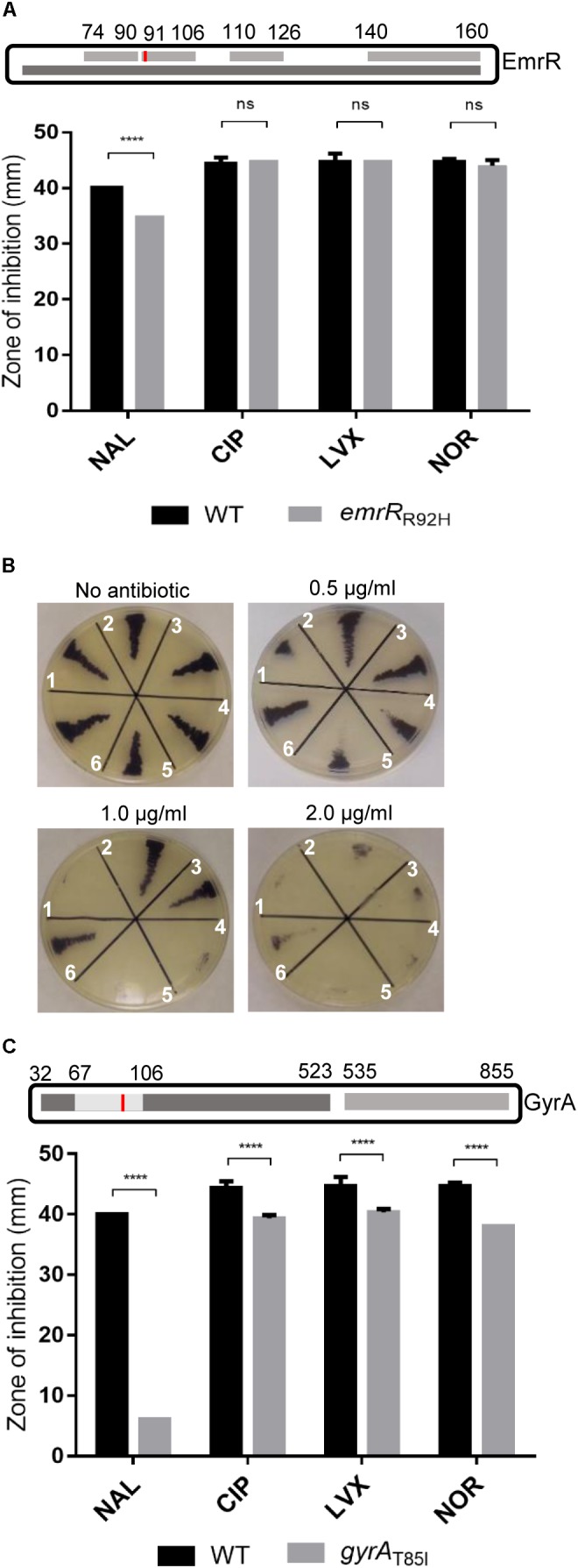
Point mutations in EmrR and GyrA confer distinct levels of quinolone resistance in *C. violaceum*. **(A)** Profile of quinolone resistance in the spontaneous mutant *emrR*_R92H_ indicated specific resistance to nalidixic acid. (Upper panel) Scheme showing the MarR domain of the EmrR protein (dark gray, entry IPR000835 from the InterPro database) with a predicted four-element fingerprint signature for the MarR family (light gray, entry PR00598 from the PRINTS database). Substitution of arginine for histidine at position 92 found in the mutant *emrR*_R92H_ is indicated (red bar). (Bottom panel) Disk diffusion assay performed on MH plates in triplicate with the indicated strains to verify the susceptibility to nalidixic acid (NAL), ciprofloxacin (CIP), norfloxacin (NOR), and levofloxacin (LVX). **(B)** MIC assay on LB plate with increasing concentrations of nalidixic acid in *emrR* mutant strains. (1) *C. violaceum* ATCC 12472; (2) Δ*emrR*; (3) *emrR*_R92H_; (4) WT(pMR20); (5) Δ*emrR*(pMR*emrR*); (6) Δ*emrR*(pMR20). **(C)** Point mutation in *gyrA* conferred resistance to multiple quinolones. (Upper panel) Schema locating the mutation in threonine 85 (red bar) of the spontaneous mutant *gyrA*_T85I_ within the QRDR region (amino acids 67–106) of GyrA. Domains of GyrA are indicated (DNA topoisomerase N and C-terminal domains indicated in dark and light gray). (Bottom panel) Disk diffusion assay for *gyrA*_T85I_ mutant with NAL, CIP, NOR, and LVX as stated in A. EmrR and GyrA proteins in the scheme are not in scale. *P*-values were determined by two-way ANOVA Sidak’s multiple comparisons test: ^∗∗∗∗^*P* < 0.0001.

To evaluate whether the emergence of nalidixic acid-resistant mutants can occur by point mutation in *emrR*, we isolated spontaneous mutants from *C. violaceum* wild-type cultivated in LB with increasing concentrations of nalidixic acid (0.5–64 μg/ml), and determined the MIC of these mutants (Table 3). DNA sequencing revealed that three isolates with MIC of 4 μg/ml had two types of mutation in *emrR* (substitution and deletion), while for the 25 remaining isolates sequenced the mutations occurred in *gyrA*, a hotspot gene for mutations that confer quinolone resistance (Giles et al., 2004). We selected two spontaneous mutants, *emrR*_R92H_ and *gyrA*_T85I_, for further characterization (Figure 3). In *emrR*_R92H_, histidine replaced arginine at position 92 of EmrR. As this substitution arose in the second (position 91–106) of a four-element fingerprint signature for the MarR family predicted in EmrR (Figure 3A) and the levels of the protein EmrR were increased in the *emrR*_R92H_ strain probably by loss of self-repression of the regulator (Figure 1B), we suggest that this point mutation affects the DNA binding activity of EmrR. In fact, *emrR*_R92H_ displayed the same antibiotic resistance profile observed for the null mutant Δ*emrR*, namely increased resistance to nalidixic acid, but not to other quinolones (Figures 3A,B). Although all point mutations in *gyrA* mapped within the quinolone resistance determining region (QRDR) of GyrA (position 67–106), the substitution of threonine for isoleucine at position 85 of GyrA had particular impact on antibiotic resistance, as the *gyrA*_T85I_ mutant showed the highest level of resistance to nalidixic acid, and presented cross resistance to other quinolones, such as ciprofloxacin, norfloxacin, and levofloxacin (Table 3 and Figure 3C). These results indicate that point mutations in EmrR and GyrA are related to the emergence of quinolone resistance in *C. violaceum*.

**Table 3:** Point mutations and amino acid changes in *emrR* and *gyrA* genes associated with increased resistance to nalidixic acid in *C. violaceum*.

Gene	Amino acid position	Nucleotide change	Amino acid change	No. of mutants	MIC^a^ Nalidixic acid (μg/mL)
*gyrA*					
	84	G**A**T→G**G**T	Asp→Gly	4	16
		**G**AT→**A**AT	Asp→Asn	2	16
	85	A**C**C→A**T**C	Thr→Ile	15	512
	89	G**A**C→G**G**C	Asp→Gly	4	16
*emrR*					
	92	C**G**C→C**A**C	Arg→His	2	4
	145/146	T**TCGA**A→TA	Phe/Glu del	1	4

^a^*MIC values determined by streaking strains on LB plates with increasing concentrations of nalidixic acid (MIC 1 μg/mL for WT under this condition)*.

### The EmrR Regulon Comprises Mostly EmrR-Repressed Genes, Including Other Genes Related to Transport Besides *emrCAB*

**FIGURE 4:**
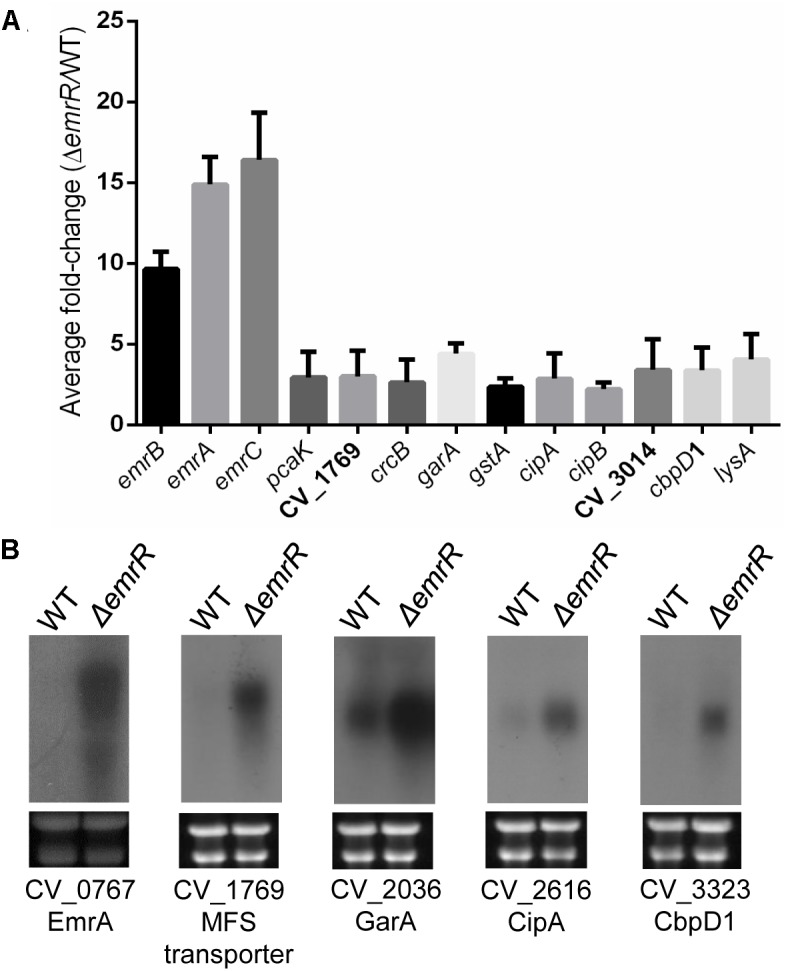
The EmrR regulon includes mainly EmrR-repressed genes. **(A)** Data from DNA microarray analysis for selected EmrR-repressed genes are shown as average ratios with standard deviations from three biological replicates comparing transcriptome from *C. violaceum* ATCC 12472 with Δ*emrR* mutant. For a complete list of the genes with altered expression levels, see Supplementary Table S4. **(B)** Northern blot assays validated several genes as members of the EmrR regulon. Total RNA extracted from *C. violaceum* ATCC 12472 and Δ*emrR* mutant were probed for the indicated genes. Levels of rRNA indicated equal RNA loading (bottom panels).

To identify the full repertoire of genes regulated by EmrR, we compared, by DNA microarray analysis, the transcriptome of the wild-type and Δ*emrR* mutant strains grown in LB medium, at log phase. From this analysis, 22 genes were upregulated and 14 genes were downregulated in the Δ*emrR* mutant compared to wild-type strain (Supplementary Table S4), indicating that EmrR acts mostly as a transcription repressor. The EmrR-repressed genes discussed in the text are shown in Figure 4A, and most of them were validated by Northern blot (Figure 4B) and EMSA (see the next item). Among the genes upregulated in the Δ*emrR* mutant, CV_0766 (*emrB*), CV_0767 (*emrA*), and CV_0768 (*emrC*) showed more than ten-fold increase in expression in the mutant. These genes compose the operon *emrCAB*, which encodes the putative MFS-type efflux pump EmrCAB (Figure 1A). However, other genes encoding putative transport proteins were upregulated (*pcaK*, CV_1769, *crcB*, CV_3014), including three MFS-type transporters. Interestingly, some genes from a pathogenicity island required for *C. violaceum* virulence (*cipA*, *cipB*) (Miki et al., 2010), and genes related to the response of *C. violaceum* to oxidative stress (*garA*, *gstA*) (Previato-Mello et al., 2017) were also upregulated in the Δ*emrR* mutant (Supplementary Table S4 and Figure 4). These data suggest that EmrR acts as a repressor of several putative transporters, including the EmrCAB efflux pump, and also regulates genes involved in other processes (virulence and oxidative stress).

### EmrR Acts as a Direct Transcription Repressor in *C. violaceum*

**FIGURE 5:**
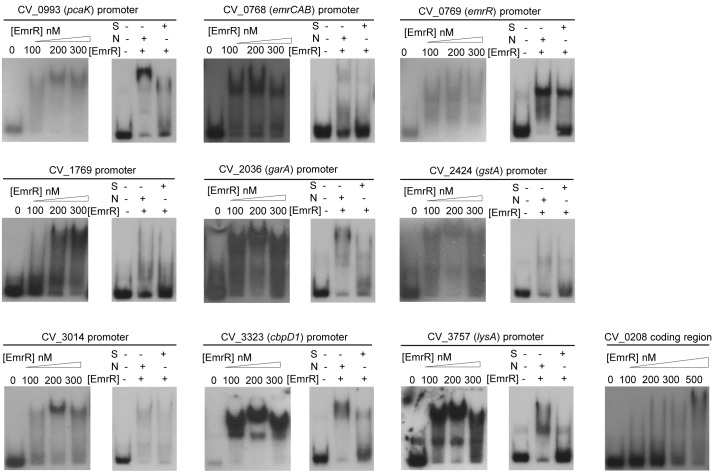
EmrR acts as a direct transcription repressor. EMSA assays with labeled probes containing the promoter regions of the indicated EmrR target genes, incubated with different concentrations of purified EmrR protein (100, 200, and 300 nM EmrR). For all cases, competition assays were performed (right panels) using 200 nM EmrR in the presence of 30-fold excess of unlabeled fragments of the same region (S) or the coding region of CV_0208 (N) as competitors. Differences in some competition assays suggest that EmrR has distinct affinities for its binding sites. A control assay performed with the coding region of CV_0208 indicated that at 500 nM, EmrR bound in a nonspecific manner.

Most MarR family transcription factors act as repressors by direct binding in the promoter region of their target genes (Perera and Grove, 2010). Thus, we employed EMSA to determine whether EmrR acts as a repressor by direct interaction with promoters of the EmrR-repressed genes. After purification of EmrR as a Histag recombinant protein with high degree of purity and homogeneity (Supplementary Figure S2), the labeled DNA fragments for promoter region of nine selected genes were incubated with increasing concentrations of EmrR. For all probes, the shift was first observed at 100 nM EmrR, whereas for a non-specific probe, the shift occurred only at 500 nM EmrR (Figure 5). Competition assays using specific and non-specific unlabeled DNA confirmed the specificity of EmrR binding (Figure 5, right panels). These data confirm that EmrR directly repressed these genes, including the *emrR* gene itself, as well as the operon *emrCAB*.

### Nalidixic Acid Resistance and Decreased Violacein Production in *emrR* Mutants Are Due to Derepression of the Efflux Pump EmrCAB

**FIGURE 6:**
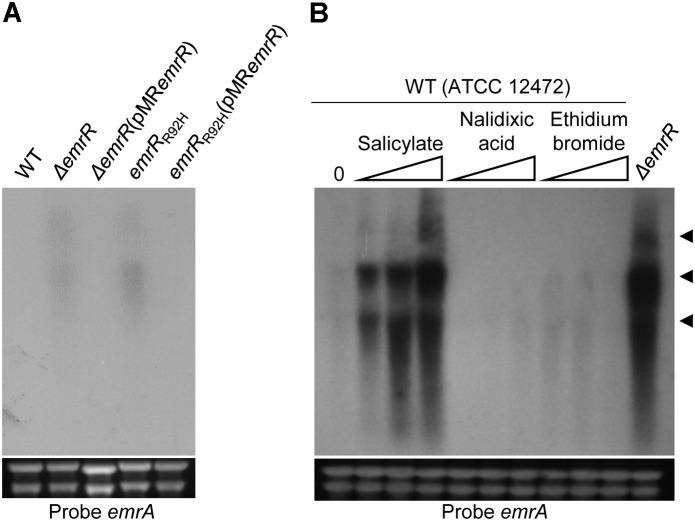
Nalidixic acid resistance in *emrR* mutants correlated to overexpression of the efflux pump EmrCAB. **(A)** Northern blot analysis using total RNA from the indicated strains grown in LB medium revealed that null or point mutation in *emrR* causes overexpression of *emrCAB*. **(B)** Salicylate caused derepression of *emrCAB*. Northern blot assay was performed using total RNA extracted from the wild-type strain ATCC 12472 before (0) or after exposure for 10 min to salicylate (0.1, 1.0, and 10 mM), nalidixic acid, and ethidium bromide (0.1, 0.2, and 0.5 mM), or RNA extracted from Δ*emrR* grown in LB medium. Levels of rRNA were used as a loading control (bottom panels). RNA samples were hybridized with a probe for *emrA*. Head arrows indicate putative isoform transcripts of the *emrCAB* operon. Distinct band intensity between **(A,B)** reflects differences in time exposure.

**FIGURE 7:**
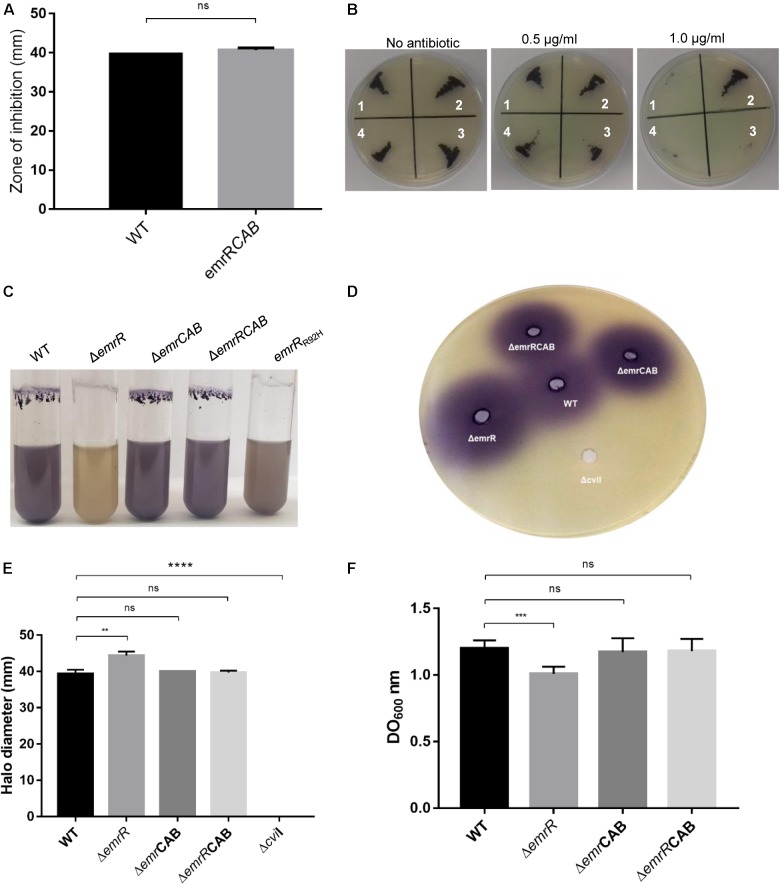
The phenotypes of increased antibiotic resistance and decreased violacein production in Δ*emrR* are relieved by deletion of *emrCAB* in this mutant strain. **(A)** Disk diffusion assays for nalidixic acid performed on MH plates in triplicate with the indicated *C. violaceum* strains. The error bars indicate standard deviations. **(B)** MIC assay on LB plate with increasing concentrations of nalidixic acid: (1) *C. violaceum* ATCC 12472; (2) Δ*emrR*; (3) Δ*emrCAB*; (4) Δ*emrRCAB*. **(C)** Production of violacein in the *C. violaceum* strains. The indicated strains were grown in LB broth at 37°C for 24 h. The emrR_R92H_ mutant produces a little more violacein than the Δ*emrR* mutant does, but both strains have decreased violacein production. **(D,E)** Quantification of violacein production in the biosensor strain Δ*cviI* (poured into the agar) stimulated by supernatants of the indicated strains revealed that Δ*emrR* accumulates extracellular AHLs. Supernatant of a Δ*cviI* culture was used as negative control. **(F)** Quantification of biofilm production by the indicated strains grown under static conditions. *P*-values were determined by *t*-test Student: ^∗∗^*P* = 0.0061; ^∗∗∗^*P* < 0.001; ^∗∗∗∗^*P* < 0.0001.

DNA microarray analysis and Northern blot assay using the wild-type and Δ*emrR* strains indicated that EmrR repressed the operon *emrCAB* (Figure 4). To further reinforce the regulation of *emrCAB* by EmrR, the Northern blot assays were performed with other strains and conditions (Figure 6). When the strains were grown in LB medium, the operon *emrCAB* was highly expressed in both the Δ*emrR* and *emrR*_R92H_ mutant strains, whereas in the wild-type and complemented strains its expression was fully repressed, suggesting that Δ*emrR* and *emrR*_R92H_ produce the EmrCAB efflux pump at high levels (Figure 6A). To test if *emrCAB* expression can increase in wild-type *C. violaceum* in response to other compounds, salicylate, ethidium bromide, and nalidixic acid were tested with Northern blot assays. Only salicylate induced the operon *emrCAB* in a dose-dependent manner (Figure 6B). To confirm whether the phenotypes of increased nalidixic acid resistance and decreased violacein production in the Δ*emrR* mutant were because this strain overexpresses *emrCAB*, we constructed and characterized Δ*emrCAB* and Δ*emrRCAB* mutant strains (Figure 7). Deletion of *emrCAB* in the wild-type strain had no effect on susceptibility to several antibiotics (Supplementary Figure S1), including nalidixic acid (Table 2, Supplementary Figures S1, and Figure 7B), nor altered violacein production (Figure 7C) in relation to the wild-type strain, suggesting that the absence of the EmrCAB efflux pump can be replaced by other efflux pumps. On the other hand, deletion of *emrCAB* in the Δ*emrR* background (strain Δ*emrRCAB*) restored nalidixic acid susceptibility (Table 2 and Figures 7A,B) and violacein production (Figure 7C) altered in the Δ*emrR* mutant, confirming that EmrR controls these phenotypes via EmrCAB. As violacein production is activated by AHL-mediated signaling in *C. violaceum* (Morohoshi et al., 2008; Stauff and Bassler, 2011), we thought that the absence of violacein in Δ*emrR* could be related to the secretion of AHL mediated by EmrCAB. Indeed, using the biosensor strain Δ*cviI*, which does not produce AHLs nor violacein, but does produce violacein in response to exogenous AHL, we verified that Δ*emrR* accumulated extracellular AHLs (Figures 7D,E) and presented decreased biofilm formation (Figure 7F), suggesting that overexpression of EmrCAB decreases the intracellular accumulation of AHLs and of nalidixic acid.

## Discussion

In this work, we characterized the involvement of the MarR family transcription factor EmrR in antibiotic resistance and violacein production in *C. violaceum.* The mutant strain Δ*emrR* showed increased resistance to the antibiotic nalidixic acid and decreased production of violacein. Because these phenotypes were relieved after simultaneous deletion of *emrR* and *emrCAB*, we propose that EmrR acts in these cases via the MFS-type efflux pump EmrCAB. Moreover, we isolated spontaneous mutant strains in increased nalidixic acid concentration that had point mutations in *emrR* and displayed phenotypic characteristics similar to the null mutant Δ*emrR*. This suggests that point mutation in *emrR* can contribute to emergence of antibiotic resistance. Null deletion or point mutation in *emrR*, or molecules that attenuate EmrR repression, such as salicylate, all caused overexpression of the EmrCAB efflux pump, indicating that EmrR confers resistance to nalidixic acid and controls violacein production by acting as a repressor of the *emrCAB* operon. Thus, at high levels, EmrCAB could export nalidixic acid, and AHL molecules involved in activating violacein biosynthesis. Finally, transcriptome analysis and validation by biochemical assays demonstrated that EmrR repressed other putative transporters besides the *emrCAB* operon, by directly binding to the promoter regions of these genes.

Overexpression of efflux pumps as consequence of mutation in transcription factors is associated with antibiotic resistance in many bacteria (Fernandez and Hancock, 2012; Blair et al., 2015). Although the emergence of antibiotic-resistant strains due to point mutations in genes encoding repressors of the MarR family has been reported (for instance MexR and MepR) (Higgins et al., 2003; Birukou et al., 2013), this is the first description of this mechanism occurring in EmrR, at least under laboratory selection conditions. Indeed, most of the spontaneous nalidixic acid-resistant mutants presented point mutation in the QRDR region of GyrA, a mechanism well-documented in the literature (Redgrave et al., 2014). Our results also suggest that the arginine 92 is critical for the DNA binding activity of EmrR, as *emrR*_R92H_ presented a phenotype similar to Δ*emrR*. Considering that we sequenced only *emrR* and *gyrA* genes, we can not exclude the occurrence of mutation in other topoisomerase genes (*parC*, *gyrB*) as contributing for quinolone resistance in *C. violaceum*.

Our data indicated that the *emrR* mutant strain showed increased resistance only to nalidixic acid, among the 24 antibiotics tested. This limited effect, also found for *emrR* mutants of *E. coli* and *S. maltophilia*, has been attributed to the fact that the EmrR-regulated efflux pump is specific for hydrophobic compounds (Lomovskaya and Lewis, 1992; Lomovskaya et al., 1995; Huang et al., 2013). Interestingly, our findings indicated that EmrCAB seems to have a role in exporting long-chain AHL molecules, which are synthesized by CviI and are required to activate violacein biosynthesis in *C. violaceum* (Morohoshi et al., 2008; Stauff and Bassler, 2011). In fact, RND-type efflux pumps have been associated with the secretion of quorum sensing molecules in other bacteria (Yang et al., 2006; Chan et al., 2007; Fernandez and Hancock, 2012). In *C. violaceum*, mutation of the *emrCAB* operon in the wild-type background had no effect on antibiotic susceptibility (including nalidixic acid), suggesting that other efflux pumps could be more active in absence of *emrCAB* (perhaps other putative transporters found as members of the EmrR regulon), and that the EmrCAB efflux pump should be relevant only in conditions that increase its expression. In fact, in *S. maltophilia*, mutation of *emrCABsm* only contributed to antibiotic susceptibility when combined with mutation in *tol*C, a condition that inactivated several TolC-dependent efflux pumps (Huang et al., 2013). Thus, high expression of the EmrCAB efflux pump (as in *emrR* mutants), but not its absence, allows a clear identification of its role in antibiotic resistance.

Similarly to what we observed in *C. violaceum*, in both *E. coli* and *S. maltophilia* EmrR represses the operon *emr*R(C)AB (Xiong et al., 2000; Huang et al., 2013). However, the natural ligand of EmrR remains to be determined, or even whether the same ligand works in *emrR* of all species. Indeed, derepression of *emrCAB* by salicylate occurs in both *C. violaceum* and *E. coli* (Lomovskaya et al., 1995), but was not observed in *S. maltophilia* (Huang et al., 2013).

The scope of action of EmrR seems broader than just regulating antibiotic resistance, because our global analysis of its regulon in *C. violaceum* revealed that it repressed genes involved in processes distinct from transport (the *emrCAB* operon and other putative transporters). One of these processes can be response to oxidative stress. Indeed, we verify that an antioxidant enzyme involved in *C. violaceum* resistance to peroxide (Previato-Mello et al., 2017) was regulated by EmrR. Accordingly, in both *E. coli* and *C. violaceum* EmrR was found to be induced under oxidant conditions (Sakamoto et al., 2015; Lima et al., 2016). Thus, it will be interesting to investigate whether EmrR can act as a redox sensing regulator, as is the case for other MarR family transcription factors related to antibiotic resistance (MgrA, MexR, OspR) (Chen et al., 2011).

The mechanism of point mutation for emergence of intrinsic resistance appears be more relevant for *C. violaceum* than those involving transfer of mobile genetic elements, considering that *C. violaceum* does not seem to harbor plasmids (Vasconcelos et al., 2003) and that several clinical isolates of this bacterium were found to be resistant to antibiotics (Aldridge et al., 1988; Yang and Li, 2011; Hagiya et al., 2014; Madi et al., 2015). In fact, two recent reports described the emergence of antibiotic resistance in *C. violaceum* by point mutations in transcription factors from the TetR family that regulate RND-type efflux pumps (Banerjee et al., 2017; Evans et al., 2018). Our data demonstrating that mutation in *emrR* is a potential mechanism that can favor the emergence of resistant strains improves the understanding on the regulatory mechanisms of antibiotic resistance in *C. violaceum*, contributing to formulating better treatment strategies against this pathogen.

## Author Contributions

KB, MP-M, BB, and JB performed the experimental work. KB prepared the figures and tables. JSN contributed in study conception and data interpretation. JSN, KB, and BB wrote the manuscript. All authors participated in data interpretation and contributed to manuscript revision.

## Conflict of Interest Statement

The authors declare that the research was conducted in the absence of any commercial or financial relationships that could be construed as a potential conflict of interest.

## Footnotes

1 http://www.ncbi.nlm.nih.gov/geo/
