# Supplementary material for: EmrR-Dependent Upregulation of the Efflux Pump EmrCAB Contributes to Antibiotic Resistance in Chromobacterium violaceum
Source: Front Microbiol. 2018 Nov 15;9:2756. doi: 10.3389/fmicb.2018.02756 (PMC6249546; doi:10.3389/fmicb.2018.02756)
Supplement: Supplementary file 1 [file Data_Sheet_1.pdf]

Supplementary Material

EmrR-Dependent Upregulation of the Efflux Pump EmrCAB Contributes to Antibiotic Resistance in *Chromobacterium violaceum*

Kelly C. M. Barroso[1], Maristela Previato-Mello[1], Bianca B. Batista[1], Juliana H. Batista[1], José F. da Silva Neto[1*]

[1]Departamento de Biologia Celular e Molecular e Bioagentes Patogênicos, Faculdade de Medicina de Ribeirão Preto, Universidade de São Paulo, Ribeirão Preto, SP, Brazil

*** Correspondence:** José F. da Silva Neto, jfsneto@usp.br

Table S1: Oligonucleotide sequences.

Primer	Nucleotide sequence (5’- 3’)[a]	Purpose
Cloning		
CV0769Exp-Fw[b]	CCAGCTCATATGAGTCCAAACAAGTCCTTTTC	Fragment NdeI/BamHI 501 bp, to clone *emrR* into pET15b
CV0769Exp-Rv[b]	AATGCAGGATCCTCAGCCGCCGAGCTTGCTC	
CV0769_comp_Fw	TTGGCACTGCAGCAAATAAGCCTGCCCGTGGC	Fragment PstI/SacI 775 bp, to clone *emrR* into pMR20
CV0769_comp_Rv	TTGGCAGAGCTCCAATGATGCATGTCGGTCCG	
Construction of deletion mutant strains		
CV_0769_del1[c]	ATGGGCCCGGCAAGCTAGCCAGGATGGC	Fragment ApaI/HindIII 675 bp, to clone flanking region into pNPTS138
CV_0769_del2	TATAAGCTTCTTGTTTGGACTCATGCGGCC	
CV_0769_del3	TATAAGCTTGGCTGAGCCCGCGCTTTTTC	Fragment HindIII/BamHI 681 bp, to clone flanking region into pNPTS138
CV_0769_del4[c]	ATGGATCCCTGGGACAGCAGCCTGGACTG	
Operon_Emr_del1[c]	TACCGGAAGCTTAGGAAGTGATCCTGACCCGC	Fragment HindIII/BamHI 662 bp, to clone flanking region into pNPTS138
Operon_Emr_del2	TACCGGGGATCCATTCTGTTGGCCGGCGACGG	
Operon_Emr_del3	TACCGGGGATCCACCGAGCATGTGACCCAGTAC	Fragment BamHI/EcoRI 655 bp, to clone flanking region into pNPTS138
Operon_Emr_del4[c]	TACCGGGAATTCGGAGACGGACCGGAGTTTTTC	
CV4091-del1[c]	CCTAGCGGGCCCCAGCAGCTGGCTGCATTGCG	Fragment ApaI/HindIII 734 bp, to clone flanking region into pNPTS138
CV4091-del2	GGCCTAAAGCTTCAGTCCACCTTGTTGCAGCG	
CV4091-del3	GGCCTAAAGCTTAGGCAGGCGAAAACCGAGC	Fragment HindIII/BamHI 650 bp, to clone flanking region into pNPTS138
CV4091-del4[c]	GGCCTAGGATCCCATTCTGGTATTGGGACGCC	
EMSA		

CV0208CDS-Fw	TTGGATCCGAAGGCCAGGCGCTGCATCT	Fragment 243 bp internal to coding region of CV_0208
CV0208CDS-Rv	ATTACTGCAGCTGTCGCTGCTGCGCACGAA	
CV0993ProFw	TGGAGGACGAGGAAAGCTGA	Promoter region of CV_0093 (354 bp)
CV0993ProRv	GCGTCGGGCAAGGCGTCTAT	
CV0769del3	TATAAGCTTGGCTGAGCCCGCGCTTTTTC	Promoter region of *emr*CAB (104 bp)
CV0769_comp_Rv	TTGGCAGAGCTCCAATGATGCATGTCGGTCCG	
CV0769_comp_Fw	TTGGCACTGCAGCAAATAAGCCTGCCCGTGGC	Promoter region of *emrR* (211 bp)
CV0769del2	TATAAGCTTCTTGTTTGGACTCATGCGGCC	
CV1769ProFw	AAGGCGGAAATCCCGGTCAG	Promoter region of CV_1769 (216 bp)
CV1769ProRw	GTACCGGCCAGAGTACGGTA	
CV2036ProFw	ATGCATGGCTGTTGAGGCTG	Promoter region of CV_2036 (247 bp)
CV2036ProRv	TGCGGAAAGTGACGTTCGGT	
CV2424ProFw	ATGCCTCAGTACCAGGCTGA	Promoter region of CV_2424 (226 bp)
CV2424ProRv	CTCGAGGAACAGGGTGATCT	
CV3014ProFw	TGGGACCTGCCGTCGATGCC	Promoter region of CV_3014 (185 bp)
CV3014ProRv	GCTCATGCCGAACAGCACAT	
CV3323ProFw	TGATCGTCTCTTACCGGGCC	Promoter region of CV_3323 (311 bp)
CV3323ProRv	GCCCATGACGAGGTGCTTAA	
CV3757ProFw	ATCACCGCCCGGAACACTTC	Promoter region of CV_3757 (229 bp)
CV3757ProRv	CCATAGCGGCGTGCCGTACT	

Northern blot

NB-Fw0767	CGCCCAGACCGTGCGCCAG	Fragment 324 bp internal to CV_0767 (*emrA*)
NB-Rv0767	GTGCGCTGCAGCGCGAGCC	
NB-Fw1769	TACCTGACCTCGCAAGCC	Fragment 590 bp internal to CV_1769
NB-Rw1769	ATCTGGGCGAGGGTGATC	
NB-Fw2036	ATGTTACCACCGCCGAGC	Fragment 540 bp internal to CV_2036
NB-Rw2036	TGTCCAGCGGCACTTCGA	
NB-Fw2616	TCGAAGATCTGCGCCAGC	Fragment 528 bp internal to CV_2616
NB-Rw2616	ACAGGCTCTGTCCCACCT	
NB-Fw3323	CCAACGACGGCAAGCCGGAG	Fragment 389 bp internal to CV_3323
NB-Rw3323	GCCCGGCACCGGCGGATTCA	
Sequencing		
GYRA-QRDR-FW	ATGACCGATAACCTGTTCGCC	Fragment 419 bp, QRDR region of *gyrA*
GYRA-QRDR-RV	ATGTCGGCCAACAGCTCGTG	

[a]Underlined letters indicate the restriction enzyme recognition sites, used for cloning purposes.

[b]Primers used also to sequencing *emrR* gene in nalidixic acid spontaneous mutants.

[c]Primers used also to confirm mutant strains (pairs del1/del4).

To delete *emrRCAB*, we used primers CV_0769_del1/del2 with primers Operon_Emr_del3/del4.

Table S2: Antibiotic disks used in this work.

Antibiotic[a]	Class	Abbreviation	Quantity
Nalidixic acid	Quinolone	NAL	30 µg
Ciprofloxacin		CIP	5 µg
Levofloxacin		LVX	5 µg
Norfloxacin		NOR	10 µg
Kanamycin	Aminoglycoside	KAN	30 µg
Amikacin		AMK	30 µg
Neomycin		NEO	30 µg
Tobramycin		TOB	10 µg
Chloramphenicol	Phenicol	CHL	30 µg
Imipenem	Carbapenem	IPM	10 µg
Meropenem		MEM	10 µg
Ampicillin	Penicillin	AMP	10 µg
Amoxicillin-clavulanic acid		AMC	20/10 µg
Ticarcillin		TIC	75 µg
Cefotaxime	Cephalosporin	CTX	30 µg
Ceftazidime		CAZ	30 µg
Cefoperazone		CFP	75 µg
Cefoxitin		FOX	30 µg
Aztreonam	Monobactam	ATM	30 µg
Tetracycline	Tetracycline	TET	30 µg
Doxycycline		DOX	30 µg
Erythromycin	Macrolide	ERY	2 µg
Fosfomycin	Other class	FOF	200 µg
Rifampin	Ansamycin	RIF	5 µg

[a]Antibiotic disks purchased from BD (BBL™ Sensi-Disc™ Antimicrobial Susceptibility Test Discs).

Table S3: MIC of the Δ*emrR* mutant using eight antibiotics.

	MIC (µg/ml)[a]							
Strain	NAL	STR	TET	CHL	ERY	KAN	CTX	DOX
ATCC 12472	16	32	1	8	16	32	256	2
Δ*emrR*	64	32	1	8	16	32	256	2

[a]MIC values were determined by broth macrodilution method using MH medium. These assays were performed using at least three biological replicates. NAL, nalidixic acid; STR, streptomycin; TET, tetracycline; CHL, chloramphenicol; ERY, erythromycin; KAN, Kanamycin; CTX, cefotaxime; DOX, doxycycline.

Table S4: Identification of EmrR-regulated genes by DNA microarray analysis.

Open reading frame	Gene	Function	Fold change (Δ*emrR* /WT strain)
		**Upregulated**	
CV_0766	*emrB*	probable multidrug resistance protein (MFS transporter)	9.67
CV_0767	*emrA*	multidrug resistance secretion protein (HlyD membrane-fusion protein)	14.40
CV_0768	*emrC*	probable outer membrane multidrug resistance lipoprotein (OEP)	16.43
CV_0993	*pcaK*	4-hydroxybenzoate transporter (MFS transporter)	2.96
CV_1165		conserved hypothetical protein (tRNA_edit domain)	3.49
CV_1166		conserved hypothetical protein	2.35
CV_1182		conserved hypothetical protein (Endoribonuclease L-PSP domain)	2.73
CV_1639		conserved hypothetical protein (Glutathione-dependent formaldehyde-activating enzyme, GFA domain)	2.40
CV_1769		probable resistance protein (MFS transporter)	3.00
CV_1940	*crcB*	membrane protein (putative fluoride efflux transporter CrcB)	2.64
CV_1944	*clpB*	chaperone protein ClpB; heat-shock protein	2.35
CV_2036	*garA*	glutathione amide-dependent peroxidase	4.40
CV_2311	*pstA*	phosphoenolpyruvate-protein phosphotransferase	4.30
CV_2312	*manA*	mannose-6-phosphate isomerase	3.05
CV_2424	*gstA*	glutathione S-transferase	2.36
CV_2615	*iacP*	acyl carrier protein	2.14
CV_2616	*cipA*	invasion protein (type III secretion system effector CipA)	2.87
CV_2619	*cipB*	cell invasion protein (type III secretion system needle tip complex protein CipB)	2.22
CV_3014		probable transmembrane transport protein (MFS transporter)	3.42

CV_3323	*cbpD1*	carbohydrate-binding protein (lytic polysaccharide mono-oxygenase, LPMO domain)	3.38
CV_3324		probable Cytochrome b561	5.59
CV_3757	*lysA*	diaminopimelate decarboxylase (lysine biosynthesis)	4.06
		**Downregulated**	
CV_0027		hypothetical protein	0.47
CV_0321	*hutI*	Imidazolonepropionase (histidine degradation)	0.40
CV_0322	*hutG*	Formimidoylglutamase	0.48
CV_0323	*hutU*	urocanate hydratase	0.45
CV_0324		hypothetical protein (Lipocalin_5 domain)	0.40
CV_0325	*hutH*	histidine ammonia-lyase	0.38
CV_0769	*emrR*	transcriptional repressor *emr* operon, MarR family	0.17
CV_1218		hypothetical protein	0.34
CV_1647		hypothetical protein	0.50
CV_1803		hypothetical protein	0.48
CV_1884	*hipO*	hippurate hydrolase (Peptidase family M20/M25/M40)	0.34
CV_3259		probable sensory transduction histidine kinase	0.46
CV_3995	*cyoB*	cytochrome o ubiquinol oxidase, subunit I	0.41
CV_3996	*cyoA*	cytochrome o ubiquinol oxidase, subunit II	0.45

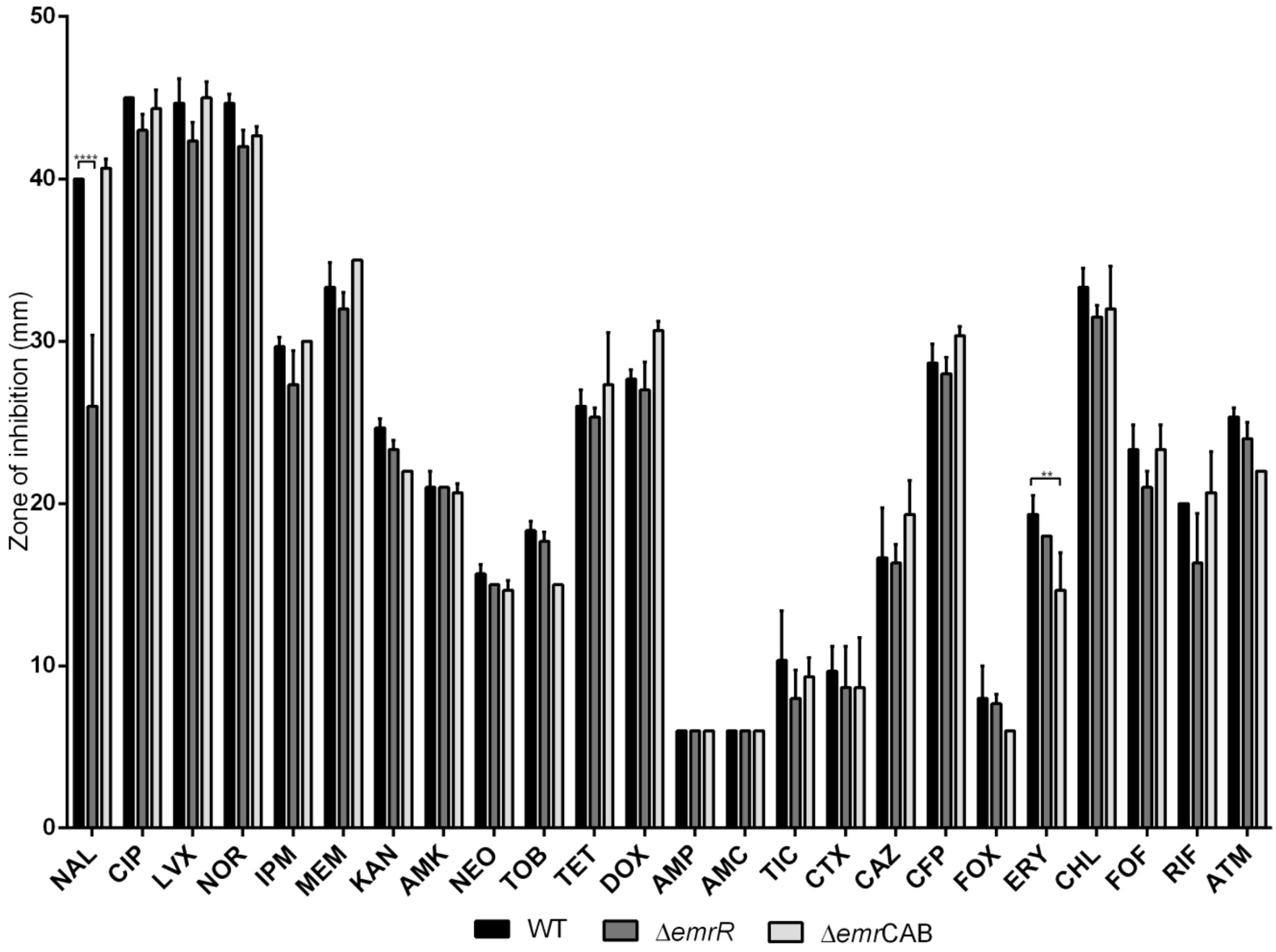

Supplementary Figure 1: Antibiogram by disk diffusion assay of the Δ*emrR* and Δ*emrCAB* mutant strains using 24 antibiotics. The assay was performed on MH plates in triplicate. The standard deviations are indicated by error bars. Nalidixic acid (NAL), Ciprofloxacin (CIP), Levofloxacin (LVX), Norfloxacin (NOR), Kanamycin (KAN), Amikacin (AMK), Neomycin (NEO), Tobramycin (TOB), Chloramphenicol (CHL), Imipenem (IPM), Meropenem (MEM), Ampicillin (AMP), Amoxicillin/Clavulanic Acid (AMC), Ticarcillin (TIC), Cefotaxime (CTX), Ceftazidima (CAZ), Cefoperazone (CFP), Cefoxitin (FOX), Tetracycline (TET), Doxycycline (DOX), Erythromycin (ERY), Fosfomycin (FOF), Rifampin (RIF), Aztreonam (ATM). The value of 6 mm (diameter of the disks) indicates absence of inhibition zone. P-values were determined by two-way ANOVA Sidak's multiple comparisons test: **** $P < 0.0001$; ** $P = 0.0021$.

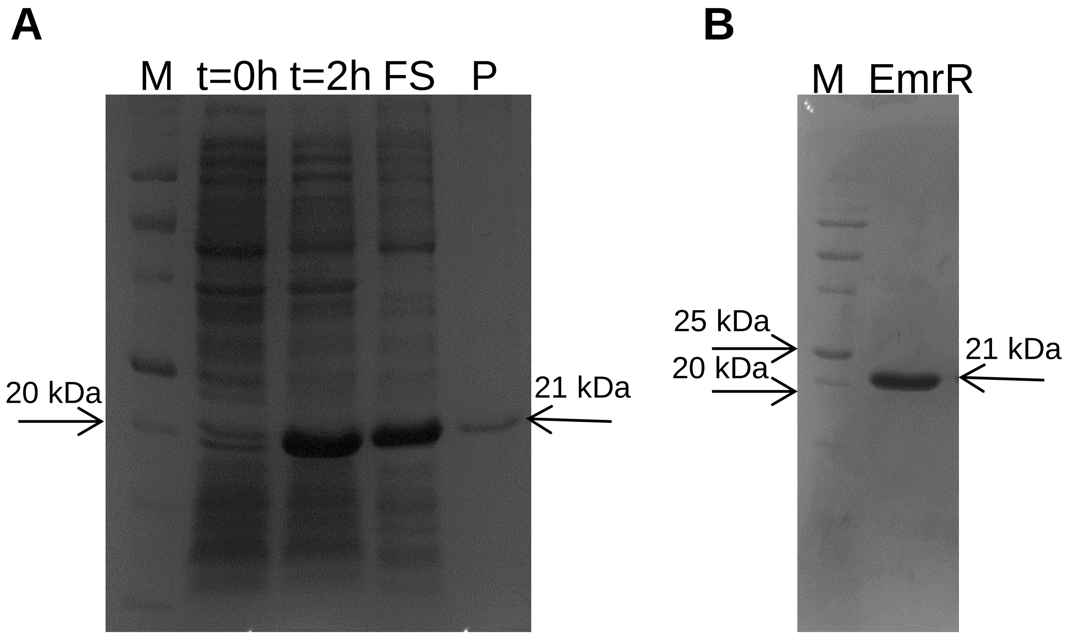

Supplementary Figure 2: Expression and purification of the recombinant EmrR protein. (A) SDS-PAGE analysis indicated that EmrR is highly induced as a soluble protein. Aliquots of cultures of *E. coli* BL21(DE3) containing pET15b(*emrR*) were collected before (t=0, not induced) and after addition of 1 mM IPTG (t=2, 2 hours). Total cell extracts (0h and 2h), soluble fractions (FS), or pellets (P) were analyzed by SDS-PAGE. (B) SDS-PAGE analysis of EmrR after purification by affinity chromatography. M: Precision Plus Protein Standard (Biorad).
